# *Salvia miltiorrhiza* in Breast Cancer Treatment: A Review of Its Phytochemistry, Derivatives, Nanoparticles, and Potential Mechanisms

**DOI:** 10.3389/fphar.2022.872085

**Published:** 2022-05-05

**Authors:** Huan Zhao, Bing Han, Xuan Li, Chengtao Sun, Yufei Zhai, Man Li, Mi Jiang, Weiping Zhang, Yi Liang, Guoyin Kai

**Affiliations:** Laboratory for Core Technology of TCM Quality Improvement and Transformation, College of Pharmaceutical Science, The Third Affiliated Hospital, Academy of Chinese Medical Science, Zhejiang Chinese Medical University, Hangzhou, China

**Keywords:** *Salvia miltiorrhiza*, breast cancer, bioactive constituent, drug combination, nanoparticle, mechanism

## Abstract

Breast cancer is one of the most deadly malignancies in women worldwide. *Salvia miltiorrhiza*, a perennial plant that belongs to the genus *Salvia*, has long been used in the management of cardiovascular and cerebrovascular diseases. The main anti-breast cancer constituents in *S. miltiorrhiza* are liposoluble tanshinones including dihydrotanshinone I, tanshinone I, tanshinone IIA, and cryptotanshinone, and water-soluble phenolic acids represented by salvianolic acid A, salvianolic acid B, salvianolic acid C, and rosmarinic acid. These active components have potent efficacy on breast cancer *in vitro* and *in vivo*. The mechanisms mainly include induction of apoptosis, autophagy and cell cycle arrest, anti-metastasis, formation of cancer stem cells, and potentiation of antitumor immunity. This review summarized the main bioactive constituents of *S. miltiorrhiza* and their derivatives or nanoparticles that possess anti-breast cancer activity. Besides, the synergistic combination with other drugs and the underlying molecular mechanisms were also summarized to provide a reference for future research on *S. miltiorrhiza* for breast cancer treatment.

## Introduction

Breast cancer is one of the most common malignancies in women worldwide. It is the dominating cause of cancer-related death after lung cancer decades ago (Freddie et al., 2018). However, according to the statistics from International Agency for Research on Cancer recently, its incidence has surpassed lung cancer and become the principal cause of cancer death in women (DeSantis et al., 2019). The morbidity of breast cancer increased at a rate of 0.3%; nearly 2,611,000 women were diagnosed in 2020 (Sung et al., 2021). The occurrence of breast cancer is often accompanied by gene mutation and/or amplification in tumor cells, such as TP53 (41% of the tumor), PIK3CA (30%), MYC (20%), PTEN (16%), CCND1 (16%), ERBB2 (13%), FGFR1 (11%), and GATA3 (10%) (Nik-Zainal et al., 2016). Based on the different molecular classifications, breast cancer can be divided into three subtypes: hormone receptor-positive/ERBB2-negative, ERBB2-positive, and triple-negative breast cancer (TNBC) (Loibl et al., 2021). Even if in the early stage, the progression of hormone-positive breast cancer could be controlled by treatment with capecitabine, tamoxifen, steroidal (exemestane), or non-steroidal (letrozole, anastrozole) aromatase inhibitors (von Minckwitz et al., 2019), etc. However, there are few therapeutic drugs for curing this condition (Blum et al., 2017). Trastuzumab plus paclitaxel-associated chemotherapy (mainly taxanes) is a first-line drug for the treatment of metastatic human epidermal growth factor receptor 2 (HER2)-positive breast cancer (Awada et al., 2016). TNBC is arduous to treat due to its high malignancy degree. Using atezolizumab monoclonal antibody improves a patient’s quality of life and prolongs survival (Schmid et al., 2018). Though chemotherapy, radiotherapy, and systemic immunotherapy increase the survival of breast cancer patients (Denkert et al., 2017; Waks and Winer, 2019), tumors often metastasize to distal organs at the late stage. Thus, it is still a challenge to cure metastatic breast cancer at present (Cardoso et al., 2018; Harbeck et al., 2019).

**FIGURE 1:**
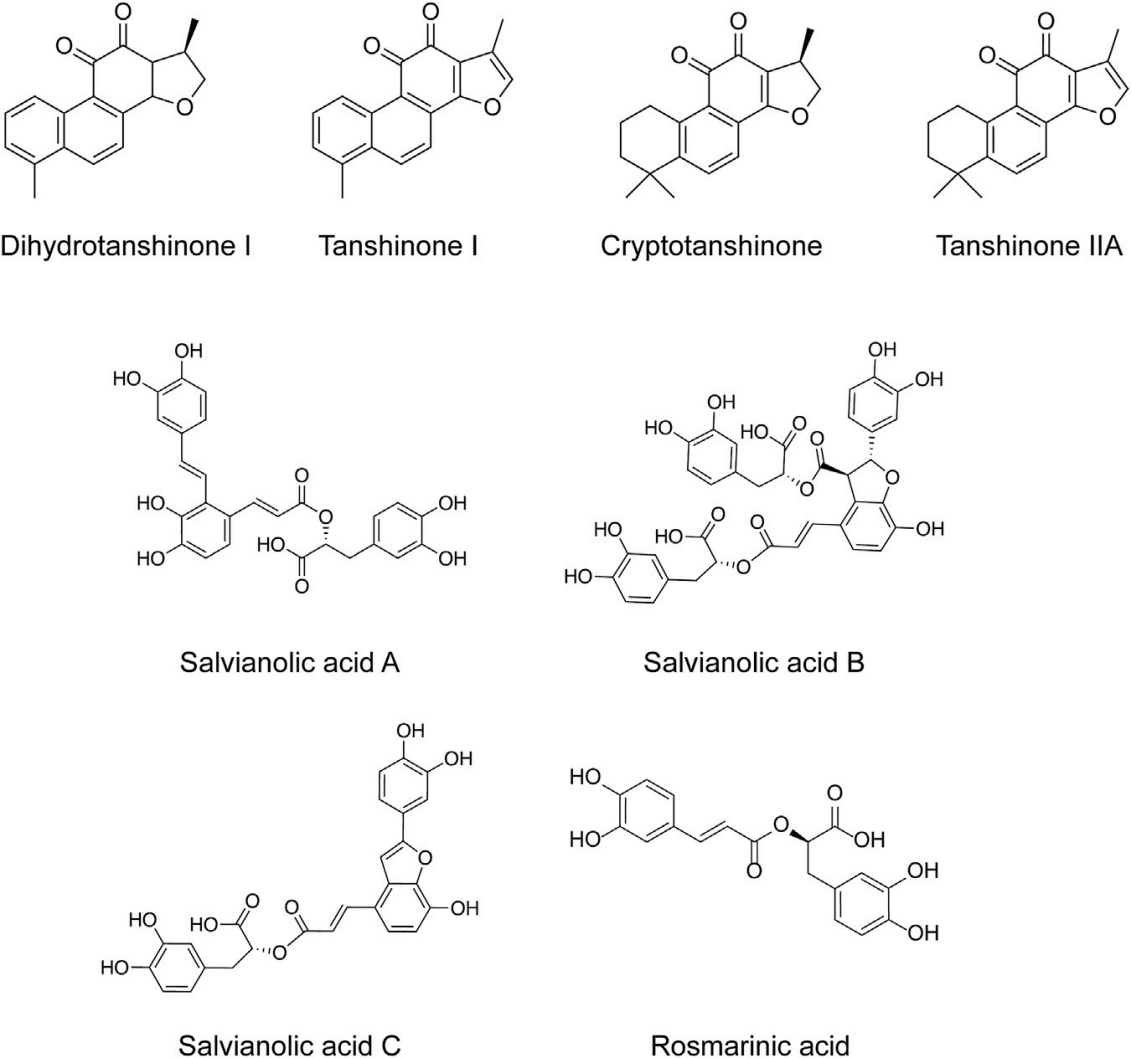
The anti-breast cancer bioactive constituents present in *S. miltiorrhiza.*

*Salvia miltiorrhiza* (*S. miltiorrhiza*), a perennial plant of the genus *Salvia*, has long been used in traditional Chinese medicine (Guo et al., 2014). It is extensively used in the management of cardiovascular and cerebrovascular disorders (Wang et al., 2018), liver diseases (Tao et al., 2013), kidney diseases (Han et al., 2021), diabetes (Zhang B. et al., 2021), and various cancers (Hung et al., 2016). The extracts of *S. miltiorrhiza* root are mainly divided into two categories, namely water-soluble compounds and liposoluble compounds. The water-soluble compounds are mainly phenolic acids represented by salvianolic acid A (Sal A), salvianolic acid B (Sal B), salvianolic acid C (Sal C), and rosmarinic acid (RA). The liposoluble diterpene quinolines are represented by tanshinones such as dihydrotanshinone I (DHT), tanshinone I (Tan I), tanshinone IIA (Tan IIA), and cryptotanshinone (CPT) (Han et al., 2008). The structures of these active compounds are shown in Figure 1.

Natural compounds from herbal medicine have gradually become mainstream drugs due to their excellent efficacy and slight side effects, as seen in the clinical application of paclitaxel in breast cancer treatment (Diéras et al., 2020). The antitumor effectiveness of the components of *S. miltiorrhiza* has been gradually excavated in these years. Based on the published literature, this review summarizes the main representative components and their derivatives or nanoparticles, pharmacological activities, and molecular mechanisms with a focus on breast cancer treatment. In addition, the possible trends and prospects are proposed, hoping to provide a reliable reference for future research.

### Main Compounds in *Salvia miltiorrhiza* With Anti-Breast Cancer Activity

#### Dihydrotanshinone I

As one of the main effective ingredients of *S. miltiorrhiza*, DHT has been extensively studied due to its anticancer, anti-inflammatory, cardioprotective, and other pharmacological activities (Chen et al., 2019). Previously, DHT has been reported to induce apoptosis and G1-phase cell cycle arrest in breast adenocarcinoma (Tsai et al., 2007). HuR is an RNA-binding protein involved in activating tumor necrosis factor (TNF), which is critical in tumor progression. Using a high throughput screening technique, DHT was found to inhibit the assembly of the HuR-RNA complex, leading to a post-transcriptional regulation of TNF mRNA stability (D'Agostino et al., 2015). Some investigators recently showed that DHT inhibited the formation of breast cancer stem cells (CSCs) and MCF-7 xenograft tumor growth in nude mice (Kim et al., 2019). Meanwhile, DHT restrained the migration and clonogenicity of highly invasive TNBC cells by inhibiting the transformation of epithelial cells into mesenchymal cells (Kashyap et al., 2021). Estrogen receptor (ER) p57 is a thiol oxidoreductase that catalyzes protein folding in the endoplasmic reticulum. DHT, as an ERp57 inhibitor, induced endoplasmic reticulum stress, triggering unfolded protein response activation and apoptosis of MDA-MB-231 cells (Shi et al., 2021).

### Tanshinone I

Tan I is another main active component of *S. miltiorrhiza*. It has significant inhibitory effects against numerous kinds of malignancies such as colon cancer (Lu et al., 2016), human endometrial cancer (Li Q. et al., 2018), breast cancer (Zheng et al., 2020), liver cancer (Zheng et al., 2020), gastric cancer (Jing et al., 2016), cervical cancer (Dun and Gao, 2019), and so on. In addition, the compound has therapeutic effects on vascular diseases (Wu Y. et al., 2019), arthritis (Wang et al., 2019), mastitis (Yang et al., 2021), and diabetes (Wei et al., 2017). Tan I was studied earlier compared with other main active substances of tanshinones. It was reported that Tan I inhibited the proliferation of MCF-7 and MDA-MB-231 cells in a dose- and time-dependent manner (TN et al., 2008). Tan I also has a potent inhibitory effect on migration and growth of MDA-MB-231 xenografts (Nizamutdinova et al., 2008). Furthermore, Tan I induced epigenetic modification of Aurora-A expression and function in the MDA-MB-231 cells (Gong et al., 2012). Another report revealed that Tan I suppressed the proliferation and induced apoptosis of MCF-7 and MDA-MB-453 cells (Wang et al., 2015). A recent study has demonstrated that Tan I inhibited the growth of MDA-MB-231 and MCF-7 cells by inducing autophagy (Zheng et al., 2020).

### Cryptotanshinone

CPT has been traditionally used in treating diabetes and cardiovascular disorders (Wang et al., 2017; Maione et al., 2018; Jia et al., 2019). Its anti-breast tumor effect has been gradually explored recently, becoming one of the hot research subjects (Wu et al., 2020a). CPT was shown to induce apoptosis of MCF-7 cells (Park et al., 2012) and inhibited the growth of xenograft tumors derived from subcutaneously transplanted MCF-7 cells in athymic nude mice (Zhou et al., 2014). Furthermore, CPT had inhibitory effects on the proliferation of ZR-75-1, MCF-7, MDA-MB-231, and MDA-MB-435 cells, and delayed the growth of ZR-75-1 breast cancer xenografts (Li S. et al., 2015). However, in another study, CPT significantly suppressed the growth of ER-positive MCF-7 cells, but had no inhibitory effect on the growth of ER-negative MDA-MB-231 cells (Pan et al., 2017). In addition, CPT induced apoptosis in SKBR-3 ER-negative but G protein-coupled estrogen receptor (GPER)-positive breast cancer cells (Shi et al., 2019). Further study confirmed that CPT did restrain SKBR-3 cell growth in a time- and dose-dependent manner (Shi et al., 2020). According to recent studies, the inhibitory effect of CPT on the proliferation and migration of MCF-7 cells is much higher than on those of MDA-MB-231 cells, suggesting its effect was associated with ER expression (Chen et al., 2020).

### Tanshinone IIA

Tan IIA is a lipophilic and the most widely explored component present in *S. miltiorrhiza*. Its derivative sodium Tan IIA sulfonate has been comprehensively used in the clinic (Ji et al., 2017; Li et al., 2017; Mao et al., 2019). Tan IIA showed numerous pharmacological effects such as anti-atherosclerosis (Lu et al., 2021), protection of cardiomyocytes and cardiac function (Gao et al., 2019; Zhang et al., 2019), improvement of diabetic osteoporosis (Ma et al., 2019; Zhang et al., 2020), repair of acute blunt skeletal muscle injury (Wang et al., 2020) and tibia cartilage dysplasia (Yang et al., 2019), protection against oxidative stress-induced myocardial cell injury (Yang G. et al., 2020), reduction of endometriosis (Chen and Gong, 2020) and traumatic brain injury (Huang et al., 2020), anti-allergy (Heo and Im, 2019), prevention of nonalcoholic fatty liver (Gao et al., 2021), mastitis (Yang et al., 2021), arthritis (Wang et al., 2019), and cerebral ischemia (Tang et al., 2019). As per the anti-tumorigenic potential of Tan IIA, it inhibited the growth of colorectal cancer (Xue et al., 2019; Liu et al., 2021), gastric cancer (Xu Z. et al., 2018), cervical cancer (Tong et al., 2020), laryngeal cancer (Xu H. et al., 2018), nasopharyngeal cancer (Wang et al., 2021), and ovarian cancer (Li N. et al., 2018).

Initially, Tan IIA was found to inhibit the proliferation of MDA-MB-231 cells (Su and Lin, 2008). Subsequently, its inhibitory effect on the proliferation and xenograft tumor growth of breast cancer MCF-7 cells were reported; its inhibitory effect was superior to tamoxifen, a clinical drug used for breast cancer treatment (Lu et al., 2009). Furthermore, Tan IIA induced mitochondrial dysfunction and apoptosis in MDA-MB-231 cells by targeting the PI3K/Akt pathway (Won et al., 2010). Further studies demonstrated that Tan IIA had a significant inhibitory effect on the growth of tumors derived from MDA-MB-231 cells implanted into the SCID female mice model (Su et al., 2012). The anti-proliferative effect of Tan IIA was also confirmed in another breast cancer BT-20 cells, as evidenced by increased the sub-G1 phase cells (Yan et al., 2012). Moreover, Tan IIA could significantly inhibit breast CSCs formation and conspicuously control the tumor growth in an MCF-7 xenograft mouse model (Lin et al., 2013). In another study, the anti-carcinogenic effect of Tan IIA was compared in MCF-7 and MDA-MB-231 cells. It showed that the inhibitory effect of Tan IIA on the growth of MCF-7 cells was superior to that of that of the latter cell line (Vanessa et al., 2014). Moreover, Tan IIA improved hypoxia-induced adriamycin resistance in breast cancer cell lines (Fu et al., 2014). It inhibited breast cancer cell growth and angiogenesis in xenograft nude mice under hypoxia and aerobic conditions (Li G. et al., 2015). Tan IIA exerted an anti-androgen effect, and thereby inhibited the growth and induced the apoptosis of T47D breast cancer cells (Zhao et al., 2015).

### Salvianolic Acids

Salvianolic acids are water-soluble components in *S. miltiorrhiza*. It principally includes Sal A, Sal B, Sal C, and RA. The efficacies of the first three in the treatment of cardiovascular diseases have been confirmed in the clinic (Wu et al., 2020b). Interestingly, the functions of these three components are similar to some extent but distinct from one another. Sal A was found to be effective in treating pulmonary hypertension (Chen et al., 2016), reducing renal injury (Zhang H. et al., 2018), ameliorating allergy (Heo and Im, 2019), cerebral ischemia (Song et al., 2019), and improving diabetic peripheral neuropathy (Xu et al., 2020). Sal B exerted protective activity against liver and oral mucosa fibrosis (Jiang et al., 2013; Chen et al., 2016), TNBC (Sha et al., 2018), cardiac arrest (Ji et al., 2020), intervertebral disc degeneration (Dai et al., 2021), and neurodegenerative disease (Zhao et al., 2019), as well as improved glucolipid metabolism in high fat diet-induced obesity (Bai et al., 2021). Sal C was shown to inhibit SARS-COV-2 infection (Yang C. et al., 2020) and protect against liver injury (Wu C. et al., 2019). All the three salvianolic acids could protect against myocardial infarction (Yu et al., 2017). RA as a precursor of phenolic acid also possesses pharmacological activities including antiviral, antibacterial, anti-inflammatory, and antioxidant effects (Petersen and Simmonds, 2003).

In regards to the anti-carcinogenic activities of salvianolic acids, Sal A remarkably inhibited the proliferation and induced apoptosis of MCF-7 cells. It also showed a significant tumor growth inhibitory effect in an MCF-7 xenograft tumor model. At the same time, it had less influence on the body weight of mice than adriamycin treatment (Cai et al., 2014). Furthermore, Sal A sensitized human breast cancer cells (MCF-7/PTX) to paclitaxel and inhibited migration and invasiveness of human breast cancer cells (Zheng et al., 2015). It was suggested that Sal A acts as an arginine methyltransferase inhibitor, thereby potentiating the anti-tumor effect of adriamycin in drug-resistant breast cancer xenografts (Li et al., 2016). Interestingly, Sal A found in another plant *Thymus carnosus Boiss* also showed growth inhibitory activity in MCF-7 and BT474 cells (Martins-Gomes et al., 2018). There was a similar case in which Sal B could induce apoptosis of MCF-7 cells in a certain time- and dose-dependent manner (Quan et al., 2019). Sal B exerted its antitumor activity at least partially by promoting ceramide accumulation and ceramide-mediated apoptosis which was attributable to its inhibition of glucosylceramide and GM3 synthases expression, independently of ERα. It was pointed out that Sal B could act as a promising therapeutic candidate against TNBC (Sha et al., 2018). Furthermore, Sal B remarkably reduced the tumor volume and increased the median survival rate in mice injected with Ehrlich solid cancer cells. It decreased the levels of oxidative stress marker (malondialdehyde) and increased plasma levels of antioxidant marker (glutathione, GSH) (Katary et al., 2019). Interestingly, it was demonstrated that RA could dose-dependently inhibit the migration of MDA-MB-231BO bone-homing, MCF-7, MDA-MB-231, and MDA-MB-468 breast cancer cells (Xu et al., 2010; Juskowiak et al., 2018; Messeha et al., 2020; Yahia Darwish et al., 2020). In addition, RA significantly improved the therapeutic effect of paclitaxel in an Ehrlich’s ascites carcinoma suspension-induced breast cancer mouse model (Mahmoud et al., 2021).

### Anti-breast Tumor Mechanisms of the Main Compounds of *Salvia miltiorrhiza*

#### Induction of Apoptosis

Programmed cell death or apoptosis of cancer cells has been the mainstream of cancer research for the past decades (Carneiro and El-Deiry, 2020). At the present stage, the primary apoptosis research focuses on the mitochondrial pathway (Han et al., 2018) which is regulated by pro-survival members (Bcl-2, Bcl-xl, Bcl-w, etc.) and pro-apoptotic proteins (Bax, Bak, Bad, Bim, etc.). The release of cytochrome *c* from mitochondria induces caspase activation, promoting apoptotic body formation and cell apoptosis (Martinou and Youle, 2011).

An early study showed that DHT induced apoptosis of breast cancer cells *via* a mitochondrial-related apoptosis pathway. This was achieved by reducing the level of anti-apoptotic protein Bcl-xl and mitochondrial membrane potential and increasing cytochrome *c* release. In addition, DHT triggered the cleavage of Caspase-9, Caspase-3, and Caspase-7. Meanwhile, pretreatment of cells with a pan-caspase inhibitor blocked DHT-induced apoptosis, corroborating the involvement of the Caspase-3-dependent pathway (Tsai et al., 2007). CPT induced apoptosis by stimulating CHOP-mediated endoplasmic reticulum stress, promoting ROS production and PARP cleavage (Park et al., 2012). CPT could interact with GPER, thereby blocking the PI3K/Akt signal transduction pathway (Shi et al., 2019). Furthermore, the apoptosis-promoting effect of CPT in SKBR-3 cells was reversed by silencing GPER (Shi et al., 2020). In addition, CPT inhibited STAT3, p-STAT3^Ser727^, p-STAT3^Tyr705^, c-Myc, and Bcl-2 expression in spontaneous Tientsin Albino two breast cancer mice (Du et al., 2020). Likewise, Tan I induced apoptosis of MCF-7 and MDA-MB-231 cells through activation of Caspase-3, down-regulation of Bcl-2, and up-regulation of Bax (TN et al., 2008). Similarly, Tan IIA induced apoptosis of MDA-MB-231 cells through up-regulation of Bax and Caspase-8 and inhibition of Bcl-2 (Su and Lin, 2008). It showed a similar pro-apoptotic effect in MCF-7 cells. Tan IIA induced apoptosis of MCF-7 and MDA-MB-231 cells by up-regulating Caspase-3, Bax, and down-regulating Bcl-2 (Vanessa et al., 2014) and P53 (Lu et al., 2009). The expression levels of p65 and Caspase-3 in the tumor tissues of Tan IIA-treated MDA-MB-231 xenografts were significantly lower than those of the tumor control group (Su et al., 2012). In addition, Tan IIA induced apoptosis of BT-20 breast cancer cells. The mechanism involves endoplasmic reticulum stress which was accompanied by increased expression of Caspase-12, GADD153, cleaved-Caspase-3, p-JNK, p-p38, Bax, and CHOP with concomitant decreases in Bcl-2, Bcl-xl, and p-ERK (Yan et al., 2012).

**FIGURE 2:**
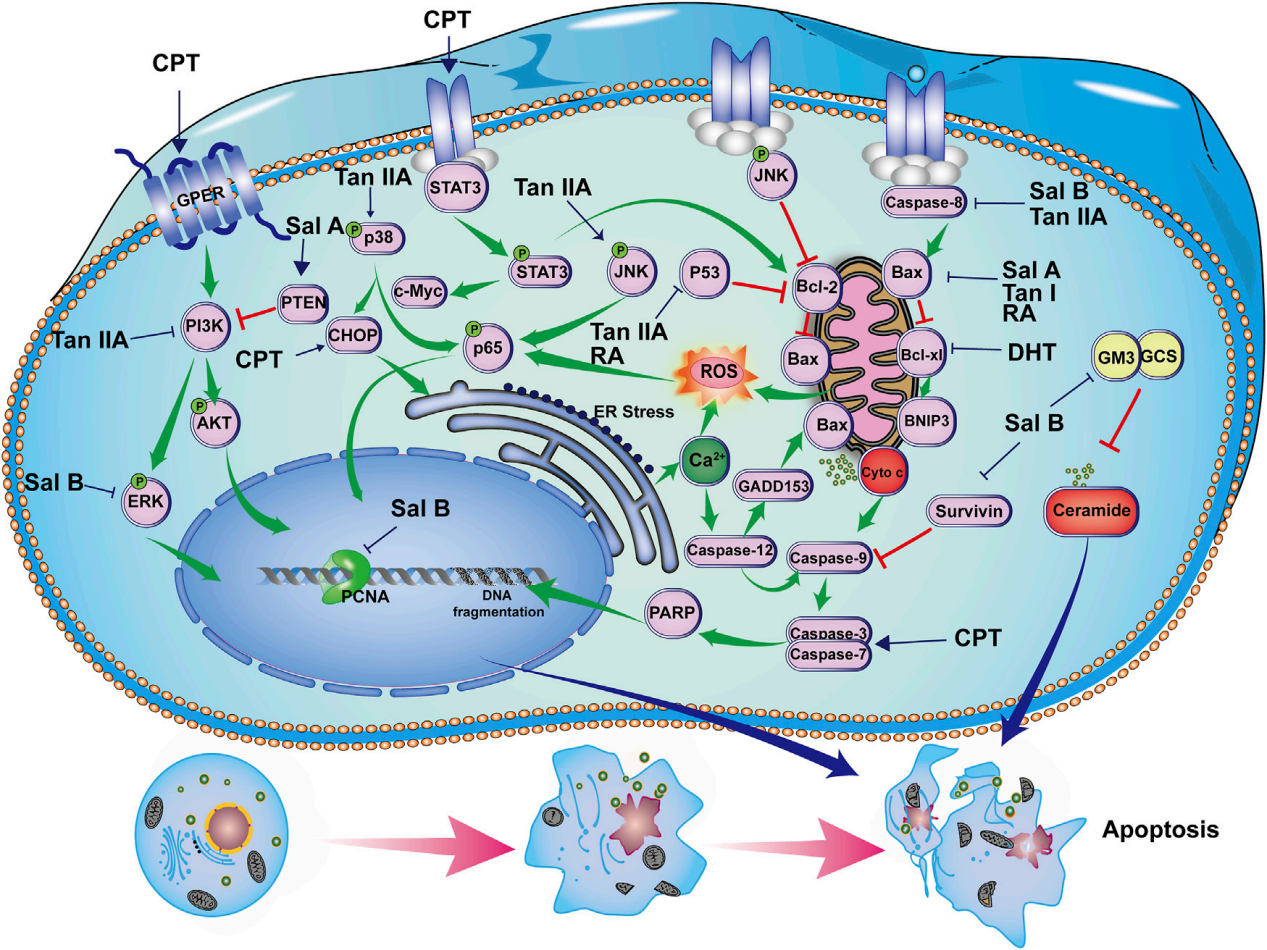
Induction of apoptosis by bioactive constituents of *S. miltiorrhiza.* CPT induces apoptosis through GPER/PI3K/Akt and STAT3 activation, c-Myc and Bcl-2 inhibition, PARP and Caspase-3 cleavage, and CHOP-mediated endoplasmic reticulum stress. DHT induces apoptosis through inhibiting Bcl-xl, promoting cytochrome C release, activating Caspase-3, Caspase-7, and Caspase-9 cleavage. Tan I inhibits Bcl-2,increases Bax, and Caspase-3 expression. Tan IIA induces apoptosis by inhibiting p53, p-ERK, Bcl-2, and PI3K/Akt pathway and increasing Bax, Caspase-3, p-p38, p-JNK expression, and CHOP-related endoplasmic reticulum stress. Sal A inhibits Bcl-2 and p-Akt, promotes PTEN and Bax expression, and meanwhile induces Caspase-3, Caspase-9, and PARP cleavage. Sal B inhibits Bcl-2, Bcl-xl, Survivin, and p-ERK expression, promotes Caspase-3, Caspase-8, and Caspase-9 activation, while it inhibits glucosylceramide and GM3 synthase expression, inducing ceramide-mediated apoptosis in breast cancer cells. RA induces apoptosis through down-regulating BNIP3 and Bcl2/Bax ratio and up-regulating P53, and caspase-3 expression.

As for salvianolic acids, Sal A inhibited the expression of Bcl-2 and p-Akt, promoted PTEN and Bax expression, and induced Caspase-3, Caspase-9, and PARP cleavage, leading to apoptosis in MCF-7 cells (Cai et al., 2014). Sal B up-regulated the expression of Caspase-3, Caspase-9, and Bax, and reduced the expression of Bcl-2 to promote apoptosis in MCF-7 cells (Quan et al., 2019). Sal B inhibited Bcl-xl, Survivin, and p-ERK expression, and promoted activation of Caspase-3 and Caspase-8 in MCF-7 and MDA-MB-231 cells. It also inhibited the expression of glucosylceramide and GM3 synthase, induced ceramide accumulation, and ceramide-mediated apoptosis in breast cancer cells. In the MDA-MB-231 xenograft mouse model, Sal B reduced the expression of PCNA, Bcl-xl, and Survivin (Sha et al., 2018). RA up-regulated the expression of BNIP3 in MDA-MB-231 and MDA-MB-468 cells. Noteworthily, the efficacy of RA in MDA-MB-231 cells was weak, as exhibited with an up-regulation of HRK, TNFRSF25, and BNIP3, and down-regulation of TNFRSF11B (Messeha et al., 2020). Another study indicated that RA and/or paclitaxel inhibited tumor growth in breast cancer models by increasing the levels of P53 and caspase-3 and inhibiting the Bcl2/Bax ratio (Mahmoud et al., 2021). Their regulatory effects on apoptosis are shown in Figure 2.

### Induction of Autophagy

**FIGURE 3:**
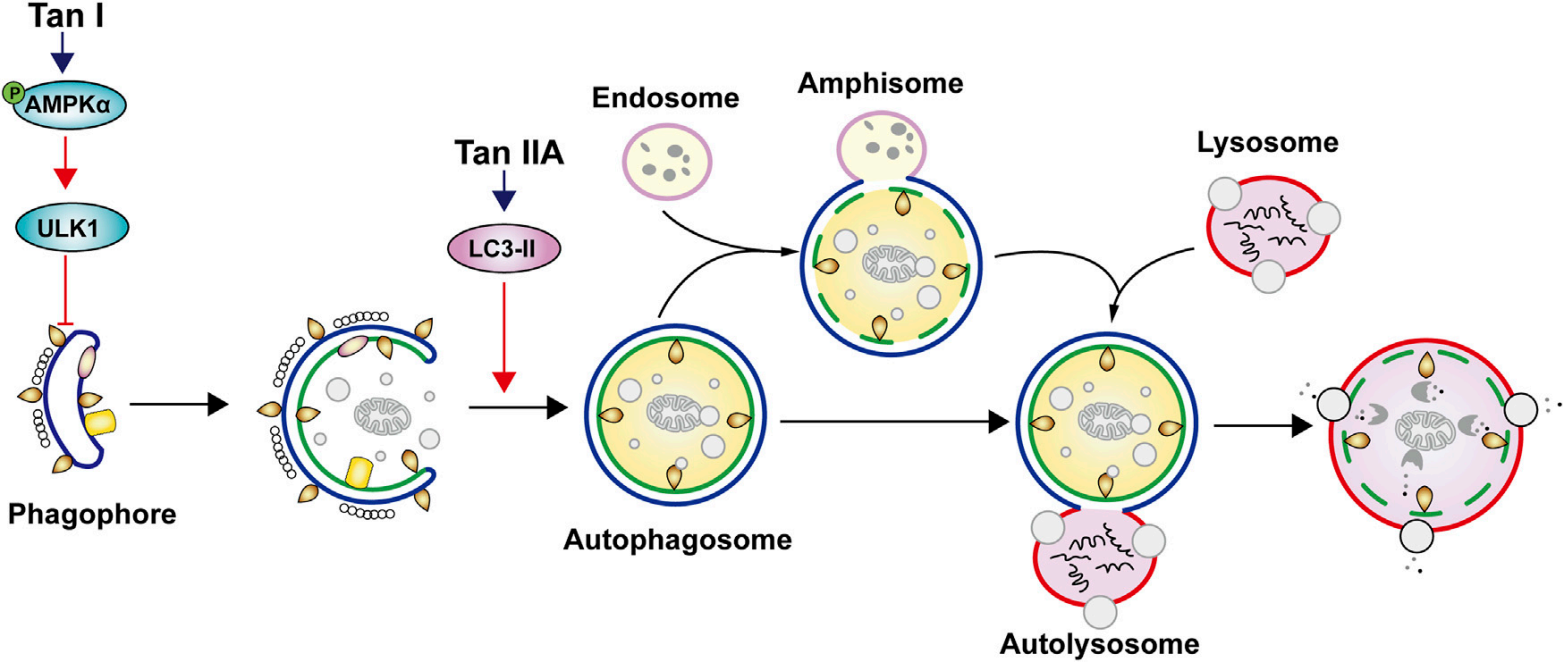
Induction of autophagy by bioactive constituents in *S. miltiorrhiza.* Tan I induces autophagy by up-regulating phosphorylation of AMPKα and its downstream ULK1 expression. Tan IIA induces autophagy by activating LC3-II expression.

Autophagy is the self-regulatory behavior of cells. It is activated to promote cell survival and tumor growth when the nutrition is deficient. Conversely, autophagy leads to cell death in the late stage (White, 2015; Levy et al., 2017). The dual nature of autophagy has attracted much attention in the scientific community (Thorburn et al., 2014). Tan I can to induce autophagy in breast cancer cells. It induced phosphorylation of AMPKα and its downstream ULK1 in MDA-MB-231 breast cancer cells (Zheng et al., 2020). Differently, Tan IIA induced autophagy in MDA-MB-231 cells by activating LC3-II expression (Yun et al., 2014). Their regulatory effects on autophagy are shown in Figure 3. So far, there is no report of salvianolic acids on autophagy.

### Induction of Cell Cycle Arrest

Since the advancement of molecular biology and modern genetics in the 1980s, the research process on malignant cells is no longer limited to the induction of apoptosis in cancer cells (Petroni et al., 2020). Cell cycle regulators such as Cyclin D, Cyclin E, Cyclin-dependent kinase 4 (CDK4), and CDK6 are discovered continuously. Recently, three inhibitors of CDK4 and CDK6 have been approved by US Food and Drug Administration (FDA) for the clinical application for hormone receptor-positive breast cancer patients (Fry et al., 2004; O'Leary et al., 2016; Gao et al., 2020).

**FIGURE 4:**
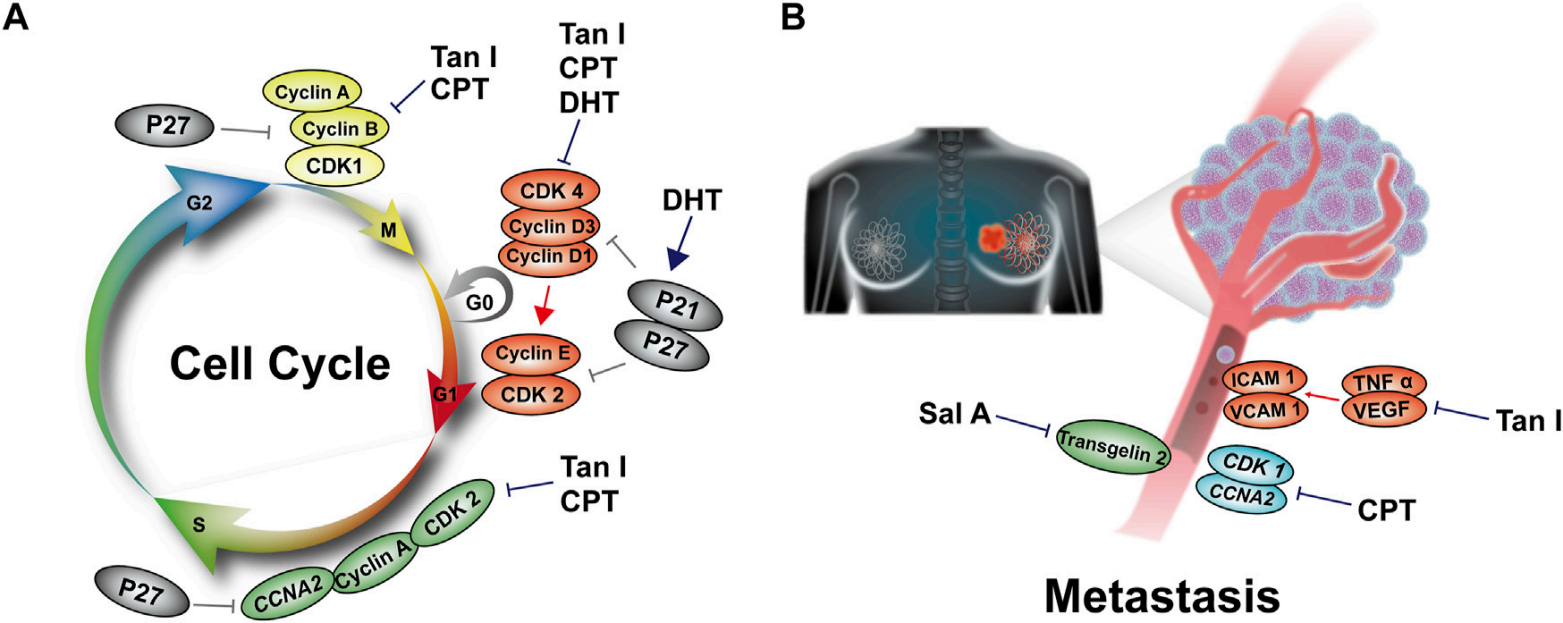
The mechanisms by which *S. miltiorrhiza* bioactive constituents induce breast cancer cell cycle and metastasis. **(A)** DHT inhibits expression of Cyclin D1, Cyclin D3, Cyclin E, and CDK4, in contrast, while increasing p21 and p27 expression, leading to G1 arrest. CPT inhibits levels of Cyclin A, Cyclin B, Cyclin D, and CDK2 expression, resulting in arrest at G1 arrest. Tan I inhibits levels of Cyclin A, Cyclin B, Cyclin D, Cyclin E, and CDK4, leading to cell cycle G1, S, or G2/M phase arrest. **(B)** Sal A inhibits Transgelin 2 expression. Tan I inhibits ICAM-1, VCAM-1, TNF-α, and VEGF expression. CPT inhibits *CDK1* and *CCNA2* gene expression, resulting in the suppression of breast cancer metastasis.

DHT could block breast cancer MCF-7 and MDA-MB-231 in the G1 phase. Further studies showed that it reduced the levels of Cyclin D1, Cyclin D3, and Cyclin E, which was accompanied by suppressed CDK4 kinase activity. In contrast, DHT up-regulated the CDK inhibitors p21 and p27 expression (Tsai et al., 2007). CPT played an anti-proliferative role in blocking cell cycle G1 phase progression through down-regulating Cyclin A, Cyclin B, Cyclin D, and CDK2 expression in SKBR-3 cells (Shi et al., 2020). Like DHT, CPT inhibited *CDK1* and *CCNA2* gene expression in MCF-7 breast cancer cells (Chen et al., 2020). Tan I treatment inhibited the expression of Cyclin D, CDK4, Cyclin B, and p-Cdc2, leading to cell cycle G0/G1 arrest in MCF-7 cells and S, G2/M phase arrest in MDA-MB-231 cells (Gong et al., 2012). It also induced S phase arrest in MDA-MB-453 and MCF-7 cells through up-regulation of CDK inhibitors p21^Cip1^ and p27^Kip1^ (Wang et al., 2015). In addition, Tan IIA was shown to inhibit breast cancer T47D cell proliferation by inducing G0/G1 arrest (Zhao et al., 2015). RA induced S phase arrest through regulation of TNF, GADD45A, and BNIP3 expression (Messeha et al., 2020). Their regulatory effects on the cell cycle are shown in Figure 4A.

### Inhibition of Metastasis

Though chemotherapy kills cancer cells, it is disappointed that some cancer cells may remain in tumor tissue and develop metastasis ultimately. Among various metastasis, breast-to-lung metastasis is the main reason for a patient death. Therefore, targeting metastasis is regarded as a practical therapeutic approach (Holm et al., 2016; Padmanaban et al., 2019; Wellenstein et al., 2019).

CPT exerted an inhibitory effect on the metastasis of MCF-7 and MDA-MB-231 cells by interfering with *CDK1* and *CCNA2* gene expression (Chen et al., 2020). In another study, the inhibitory effect of Tan I on breast cancer metastasis was confirmed in MDA-MB-231 xenograft nude mice. Tan I effectively inhibited TNF-α and VEGF expression, which further suppressed ICAM-1 and VCAM-1 expression in human umbilical vein endothelial cells (Nizamutdinova et al., 2008). In addition, the migration and invasion ability of MCF-7 human breast cancer cells (MCF-7/PTX) resistant to paclitaxel was remarkably hindered by Sal A treatment, which was associated with inhibition of Transgelin 2 expression (Zheng et al., 2015). Their regulatory effects on cancer metastasis are shown in Figure 4B.

### Regulation of Cancer Immunity

**FIGURE 5:**
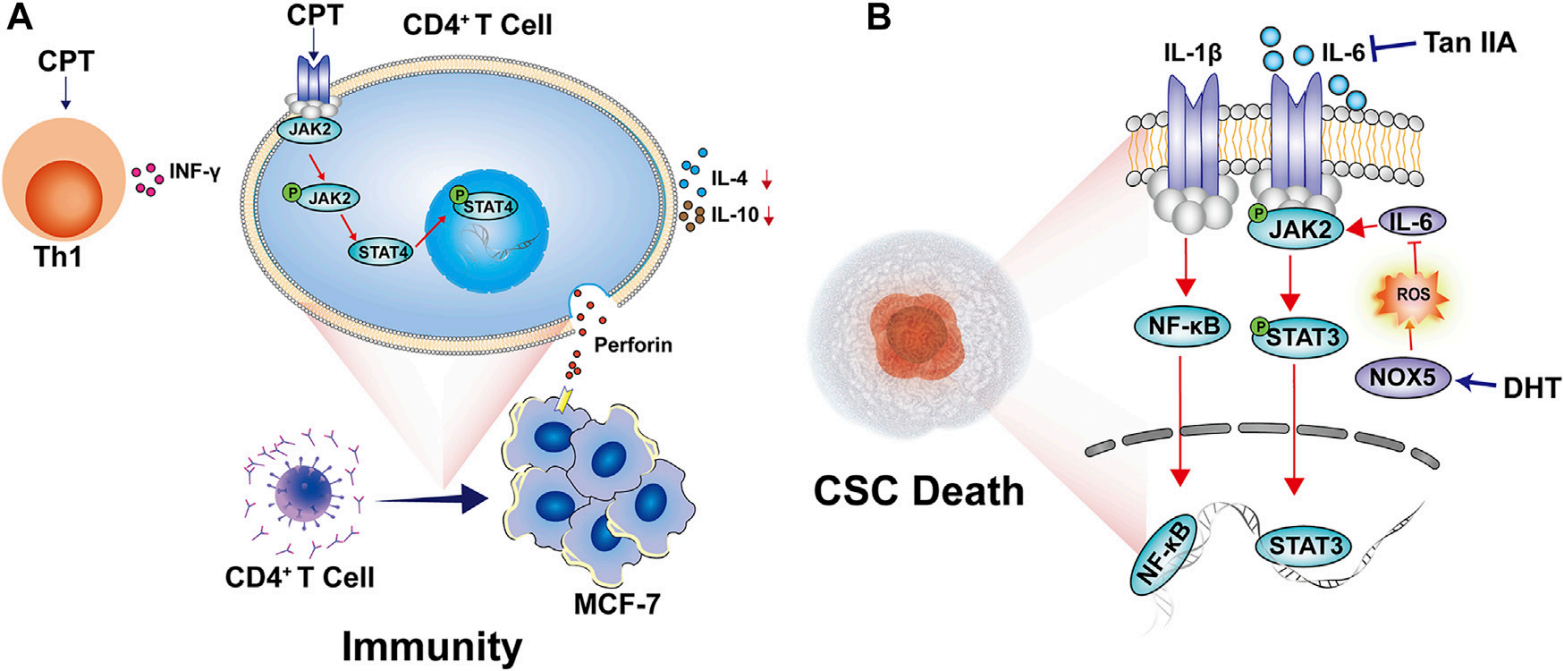
The mechanisms by which *S. miltiorrhiza* bioactive constituents modulate breast cancer immunity and CSC manifestation. **(A)** CPT enhances perforin production in CD4^+^ T cells by up-regulating phosphorylated JAK2 and STAT4, promoting immune response differentiates to Th1 type. **(B)** DHT-activated NOX5 expression promotes ROS generation which inhibits IL-6/STAT3 signaling pathway. Tan IIA inhibits the expression levels of IL-6 expression, STAT3 phosphorylation, and NF-κB p65 nucleus translation, leading to breast CSCs death.

The role of different immune cells in regulating cancer progression is becoming increasingly prominent (Wagner et al., 2019). The interaction between tumor and immune cells accounts for immunosuppression and poor prognosis (Bruni et al., 2020). Recently, immunotherapeutic drugs such as PD-1/PD-L1 and CTLA-4 are widely developed and used in clinical cancer treatment (Riley et al., 2019). The effects of bioactive constituents of *S. miltiorrhiza* on cancer immunity are not fully investigated yet. One study showed that CPT enhanced perforin production in CD4^+^ T cells by inducing the phosphorylation of JAK2 and STAT4 this modulates the immune response to Th1 type, leading to inhibition of tumor growth (Zhou et al., 2014). The regulatory effects on cancer immunity are shown in Figure 5A.

### Inhibition of Cancer Stem Cells

Breast CSCs initiate cancer cell growth in specific niches of the tumor microenvironment. The cellular and molecular components of CSCs support signaling pathways that sustain cancer cell survival, self-renewal dormancy, and reactivation (Ingangi et al., 2019). CSCs exhibit genetic, epigenetic, and cellular adaptations that confer resistance to classical therapeutic approaches. They are known to mediate metastasis and recurrence (Prager et al., 2019). Concurrently, CSCs are able to promote tumor migration by regulating epithelial-mesenchymal transformation. Due to their high drug efflux capability and anti-cancer drug resistance (Lytle et al., 2018; Lambert and Weinberg, 2021), the identification of potential CSCs targets has turned into a new therapeutic option for breast cancer (Han et al., 2020).

It was documented that DHT strikingly suppressed breast cancer CSCs and mammosphere formation (Kim et al., 2019). Meanwhile, DHT down-regulated stemness markers such as CD44^high^/CD24^low^ and aldehyde dehydrogenase and the self-renewal-related genes including *Nanog*, *SOX2*, *OCT4*, *C-Myc*, and *CD44*. DHT induced calcium and ROS production. Furthermore, DHT-activated NOX5 inhibited IL-6/STAT3 signaling and promoted CSCs death (Kim et al., 2019). Tan IIA was proven to possess potential activity to target CSCs *in vitro* and *in vivo*. It dramatically hindered the mammosphere formation and reduced expression levels of IL-6, STAT3 phosphorylation, and NF-κB p65 nucleus translation, suggesting the modulation of the IL-6/STAT3/NF-κB signaling pathway (Lin et al., 2013). Their regulatory effects on breast CSCs are shown in Figure 5B.

### Others

Estrogen and progesterone are known to increase breast cancer risk (McTiernan et al., 2005). Estrogen-induced ER transactivation and its target gene expression could be effectively reversed by CPT treatment; CPT might principally inhibit breast cancer cell growth in an ERα-dependent manner (Li S. et al., 2015). The inhibitory effect of CPT on MCF-7 breast cancer cell proliferation was associated with mTOR inhibition and dependent on ER expression (Pan et al., 2017). Hypoxia-induced adriamycin resistance and epithelial-mesenchymal transition in breast cancer cell lines. Intriguingly, Tan IIA treatment inhibited HIF-1α expression while TWIST silencing abolished its effect on cell viability (Fu et al., 2014). Tan IIA inhibited angiogenesis of breast cancer; it repressed HIF-1α expression followed by VEGF inhibition. In addition, the mTOR/p70S6K/RPS6/4E-BP1 signaling pathway was suppressed by Tan IIA possibly by inhibiting p-mTOR, p70S6K (Thr421/Ser424), RPS6 (Ser235/236, Ser240/244), and 4E-BP1 (Thr37/46) expression (Li G. et al., 2015). Sal B was shown to reduce the tumor volume in an Ehrlich solid breast cancer model. It reduced levels of plasma malondialdehyde, VEGF, TNF-α, MMP-8 and Cyclin D1, increased those of plasma glutathione, Caspase-3, and P53 (Katary et al., 2019).

## Drug Combination of the Main Compounds in *Salvia miltiorrhiza* for Breast Cancer Treatment

With the prolongation of the chemotherapy cycle, breast cancer cells are increasingly tending to acquire drug resistance. Meanwhile, a high cumulative dosage of chemotherapeutic drugs augments toxic side effects, ultimately leading to treatment failure. Moreover, CSCs have a self-renewal capacity. which hampers tumoricidal chemotherapy drugs. As a result, recurrent tumors not only are resistant to the initial treatment but also acquire a more aggressive phenotype than before (Miller-Kleinhenz et al., 2018). Combined medication is the use of two or more drugs to intervene in the disease, so as to synergize the therapeutic effect by regulating different signal pathways or target proteins (Yin et al., 2014). The pathogenesis of complex diseases such as cancer, diabetes, and cardiovascular diseases depends on complex molecular pathways and their interactions (Loscalzo et al., 2007). With the limitation of the therapeutic benefits brought by single target or single-drug therapy, the combination therapy has developed rapidly in the management of many diseases including breast cancer (Al-Lazikani et al., 2012; Iyengar, 2013).

It was reported that Sal A remarkably promoted PTEN expression through Transgelin 2, followed by inactivating PI3K/Akt signaling and increasing apoptosis, conferring enhanced the chemosensitivity of breast cancer cells to paclitaxel. It provided a clinical basis for the combined administration of Sal A with paclitaxel in breast cancer treatment (Cai et al., 2014). Co-administration of CPT (15 mm) with monomethylarsonous acid (1 mm) was found to enhance an anticancer effect against MCF-7 cells, CPT increased cancer cell sensitivity to monomethylarsonous acid treatment. The combination of monomethylarsonous acid and CPT up-regulated the expression of mitochondrial pro-apoptotic proteins, Bax and Bak, and provoked endoplasmic reticulum stress-induced by PARP-1 and Caspase-9, thereafter triggering apoptosis in MCF-7 cells (Zhang et al., 2015). Sal A was suggested as an inhibitor of arginine methyltransferase 1. Combination of Sal A (10 or 30 mg/kg) with adriamycin (8 mg/kg) inhibited the growth of adriamycin-resistant MCF-7 cells by sensitizing the cells to the anti-cancer drug (Li et al., 2016). ATP-binding cassette (ABC) transporters such as P-gp, BCRP, and MRP1 are important mediators that efflux drugs from tumor cells, resulting in drug resistance (Nobili et al., 2020). Tan IIA reduced the expression of P-gp, BCRP, and MRP1, and promoted adriamycin accumulation in adriamycin-resistant MCF-7 as well as parental cells. It effectively repressed the manifestation of breast CSCs and enhanced the chemosensitivity to adriamycin. Therefore, Tan IIA (0.02 mg/L) combined with adriamycin (2 μg/ml) was suggested as a sensitizer in breast cancer treatment (Li and Lai, 2017; Li et al., 2019). Similarly, Tan IIA (1–20 mm) inhibited the expression of the higher microtubule-associated protein Tau and resulted in increased sensitivity of MCF-7 cells to paclitaxel (5–100 mm) (Lin et al., 2018). At the same time, Tan IIA (0.5–10 μm) synergistically enhanced the antitumor effect of the Hsp90 inhibitor 17-AAG (0.001–50 μm) against MCF-7 cells by inhibiting total protein kinase C activity (Lv et al., 2018). The synergistic effect of fulvestrant (250 mg/kg, weekly, s. c.) and Tan IIA (30 mg/kg, every other day, injected *via* tail vein) combination against ER-positive breast cancer was verified in a preclinical ZR-75-1 tumor model. The combination exhibited a distinct antitumor effect than the monotherapy of fulvestrant or Tan IIA at the early time point, as monitored by 18F-FES PET/CT imaging (He et al., 2019). The nuclear translocation of β-catenin accumulation was related to drug resistance in breast cancer. Tan IIA not only dramatically inhibited nuclear translocation of β-catenin in adriamycin-resistant MCF-7 cells upon adriamycin treatment but also suppressed its expression in MCF-7 cells to some extent. Thus, the chemosensitivity of breast cancer cells to adriamycin (2 μg/ml) could be restrained by Tan IIA (20 μg/ml) by inhibiting β-catenin nuclear translocation (Li et al., 2021). In another report, gene expression of MDM2 p53 binding protein homolog and zinc finger E-box binding homeobox 1 were used to assess tumor activity compared to DOX alone in MCF-7 cells. The combination of RA (1.5, 15, 50 μm) and DOX (0.2 μm) significantly increased the expression of zinc finger E-box binding homeobox 1 gene and decreased that of MDM2 p53 binding protein homolog gene (Juskowiak et al., 2018). Interestingly, the combination of RA (100 mg/kg/day, p. o.) and paclitaxel (10 mg/kg/three times weekly, i. p.) showed anti-inflammatory and antiangiogenic effects, and the apoptosis rate was higher than that of the monotherapy. The tumor size treated with RA and paclitaxel combination showed a significant reduction. Hence, RA may increase the sensitivity of breast cancer cells to paclitaxel through the NF-κB-p53-caspase-3 pathway (Mahmoud et al., 2021). The synergistic effects of CPT, Sal A, Tan IIA, and RA for breast cancer treatment are shown in Table 1.

**TABLE 1:** The synergistic effects of active compounds in *S. miltiorrhiza* for breast cancer treatment.

Compounds	Combined compounds	Models	Dosage	Synergistic effects	Results	Ref.
CPT	Monomethylarsonous acid	MCF-7 cells	15 μm + 1 μm	Promotes apoptosis	Increased Bax, Bak, and Caspase-9	Zhang et al. (2015)
Sal A	Doxorubicin	MCF-7/DOX cells	10, 30 mg/kg + 8 mg/kg	Facilitates chemotherapy sensitivity	Decreased protein arginine methyl transferase 1 activity	Li et al. (2016)
	Paclitaxel	MCF-7/PTX cells	3, 6, 12 μM + 1,000 nM	Facilitates chemotherapy sensitivity	Inhibited PI3K/Akt pathway	Cai et al. (2014)
Tan IIA	Doxorubicin	MCF-7/DOX cells	0.02 mg/L + 2 μg/ml	Facilitates chemotherapy sensitivity	Decreased β-catenin nuclear translocation	Li et al. (2021)
	Paclitaxel	MCF-7 cells	1–20 mM + 5–100 μM	Facilitates chemotherapy sensitivity	Decreased microtubule associated protein	Lin et al. (2018)
	17-AAG	MCF-7 cells	0.5–10 μM + 0.001–50 μM	Enhances antitumor efficacy	Inhibited total protein kinase C activity	Lv et al. (2018)
	Fulvestrants	ZR-75-1 tumor xenografts	30 mg/kg/d + 250 mg/kg/w	Enhances antitumor efficacy	Decreased tumor growth	He et al. (2019)
	Doxorubicin	MCF-7/DOX cells	20 μg/ml + 2 μg/ml	Facilitates chemotherapy sensitivity and reduces toxic side effects	Inhibited PTEN/Akt pathway	(Li and Lai, 2017; Li et al., 2019)
RA	Doxorubicin	MCF-7/DOX cells	1.5, 15, 50 mΜ + 0.2 μM	Facilitates chemotherapy sensitivity	Inhibited MDM2 p53 binding protein homolog gene and increased zinc finger E-box binding homeobox 1 gene	Juskowiak et al. (2018)
	Paclitaxel	Ehrlich’s ascites carcinoma-induced Swiss albino mice	100 mg/kg/d + 10 mg/kg, 3 times/w	Enhances antitumor efficacy	Decreased tumor growth	Mahmoud et al. (2021)

Oral chemotherapy is an important strategy to treat cancer. However, due to the existence of the gastrointestinal drug barrier, the bioavailability of the most effective drugs is circumscribed. Although oral P-glycoprotein inhibitors such as cyclosporin solve this problem to some degree, it destroys the immune system (Zhang H. et al., 2021). In recent years, nanotechnology has become a hotspot because it can faultlessly solve the problem of the gastrointestinal barrier (Mei et al., 2013). Due to its low side and high curative effects, the majority of patients are more likely to receive nano-drug loading treatments and the compliance of patients is relatively good (Luo et al., 2014).

**FIGURE 6:**
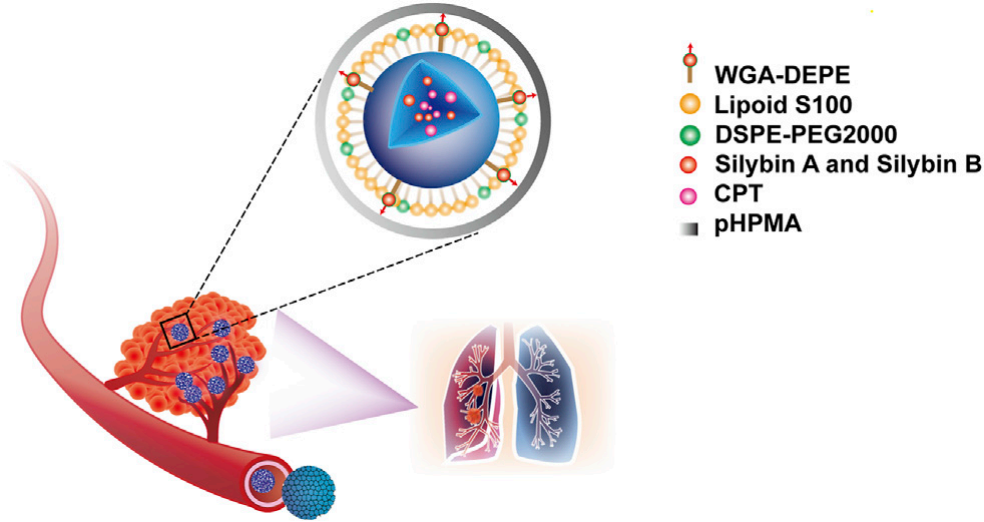
The poly-N-(2-hydroxypropyl) methacrylamide (pHPMA)-coated wheat germ agglutinin-modified lipid-polymer hybrid nanoparticles, co-loaded with silibinin and CPT (S/C-pW-LPNs).

In one study, the poly-N-(2-hydroxypropyl) methacrylamide (pHPMA)-coated wheat germ agglutinin-modified lipid-polymer hybrid nanoparticles were co-loaded with CPT and silibinin (S/C-pW-LPNs). Compared with CPT alone, S/C-pW-LPNs significantly increased 4T1 cell toxicity and inhibited cell migration and invasion. It reduced the tumor number and lung metastases in 4T1 tumor-bearing mice which were attributed to the inhibition of tumor microenvironment biomarkers such as MMP-9, TGF-β1, and CD31 (Figure 6) (Liu et al., 2020).

### Derivatives

Chemical modification is a common method to obtain derivatives with better antitumor activity. This confers increased chemotherapy sensitivity to anti-cancer treatment, higher cytotoxicity to hypoxic cancer cells, more stable characteristics, improved therapeutic index and easy access to clinical setting (Coleman et al., 1988; Shen et al., 2019).

Acetyltanshinone IIA (ATA) is a derivative of Tan IIA with higher water solubility and stronger pro-apoptotic activity in various cancer cell lines. It showed a stronger anti-proliferative and ROS production activity, especially in HER2 positive breast cancer cells. ATA treatment-induced Bax translocation, cytochrome c release, Caspase-3 activation, and apoptotic cell death, and inhibited xenografted tumor growth (Tian et al., 2010). ATA also effectively repressed the growth of ER-positive breast cancer cells. Mechanistically, ATA might achieve its effect by reducing the ERα mRNA level, and binding to ERα facilitating its degradation through the ubiquitin-mediated proteasome-dependent pathway (Yu et al., 2014). Further studies showed that ATA-induced apoptosis was related to the down-regulation of receptor tyrosine kinase/EGFR/HER2 and the downstream survival-promoting signal pathway. ATA triggered oxidative and endoplasmic reticulum stress, and AMPK activation, resulting in the inactivation of key enzymes involved in lipid, and protein biosynthesis. Intraperitoneal injection of ATA in MDA-MB-453 xenograft mice significantly inhibited the tumor growth without weight loss and any other side effects. In addition, ATA could inhibit tumor angiogenesis *in vitro* (Guerram et al., 2015). A small-size microemulsion containing sodium Tan IIA sulfonate (STS) and celastrol showed synergistic cytotoxicity to cancer cells. After sequential release in the tumor tissue, STS and celastrol-based microemulsion repaired abnormal blood vessels, reduced fibroblasts, and tumor cells, and reduced tumor size shrinkage (Qu et al., 2018). Except for modulation on tumoral blood vessels, STS also decreased collagen, cancer-associated fibroblasts, and Th2 type cytokines *in vivo* (Qin et al., 2020). The imidazole derivative analog of Tan IIA (TA12) successfully resolved the poor water solubility of Tan IIA. TA12 significantly inhibited the proliferation, migration, and invasion of MDA-MB-231 cells. In a zebrafish xenotransplantation model, TA12 also conspicuous blocked the metastasis of cancer cells in blood vessels and surrounding tissues through induction of ROS and DNA damage, leading to S phase arrest. Therefore, TA12 is expected to be an effective anti-metastasis agent (Wu et al., 2018). A synthetic derivative of CPT (KYZ3) is an effective STAT3 inhibitor. The antitumor activity of KYZ3 against TNBC cell lines was 22–24 folds higher than that of CPT, while it had little effect in normal breast epithelial MCF-10A cells. KYZ3 also inhibited TNBC cell metastasis by directly reducing MMP-9 and STAT3 levels. KYZ3 suppressed the tumor growth induced by subcutaneous implantation of MDA-MB-231 cells *in vivo* (Zhang W. et al., 2018). The effects of Tan IIA and CPT derivatives for breast cancer treatment are shown in Table 2.

**TABLE 2:** The effects of derivatives from *S. miltiorrhiza* bioactive constituents for breast cancer treatment.

Prototype	Derivatives	Structures	Characteristics	Results	Ref.
Tan IIA	ATA	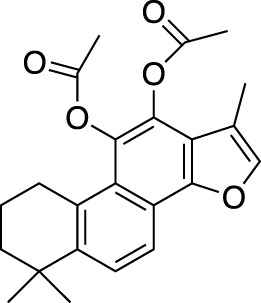	Higher water solubility, stronger pro-apoptotic activity and antitumor efficacy	Increased Bax and cleavage Caspase-3 expression	Tian et al. (2010)
				Decreased ERα expression	Yu et al. (2014)
				Triggered oxidative and ER stress and activated AMPK expression	Guerram et al. (2015)
	STS	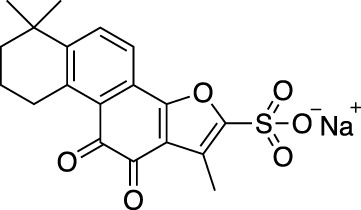	Stronger antitumor efficacy, gradient and controlled release at the tumor site	Abnormal blood vessels remodeling and reduced fibroblasts level	Qu et al. (2018)
				Photothermal triggering technology	Qin et al. (2020)
	TA12	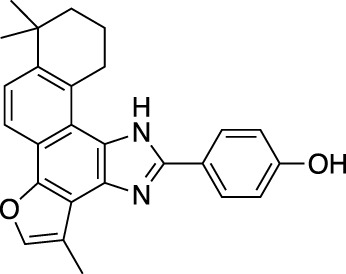	Higher water solubility, stronger pro-apoptotic activity	Activated ROS production and DNA damage leading to S arrest	Wu et al. (2018)
CPT	KYZ3	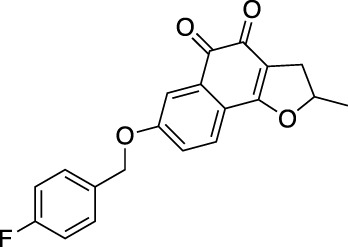	Stronger antitumor efficacy	Decreased STAT3 expression	Zhang et al. (2018b)

## Conclusion and Prospective

Breast cancer has become the most lethal cancer in the world in women. Its incidence is increasing year by year and ranked top one among women last year (Sung et al., 2021). Although the cure probability of breast cancer types other than TNBC is gradually increasing, advanced TNBC is still a largely incurable illness.

*S. miltiorrhiza* has traditionally been widely used in the management of cardiovascular and cerebrovascular diseases. With continuous exploration, its corresponding components have been continuously identified. A large number of articles on multicomponents from *S. miltiorrhiza* with regards to their therapeutic potential against breast cancer studies have been published. Their underlying mechanisms include promoting apoptosis, autophagy, cell cycle arrest, inhibiting metastasis, and regulating immunity. Various derivatives have been designed to solve the problem including poor water solubility and low efficacy. In addition, the problem of drug resistance in breast cancer was alleviated by the combination of CPT, Sal A, Tan IIA, or RA with the first-line drugs. Meanwhile, nanotechnology improved the CPT delivery system, achieved gradient release and precise targeting effect, and potentiated the anti-breast cancer activity. Until now, the clinical efficacy of these active ingredients compared with clinical drugs has not been reported yet, but they showed a therapeutic effect on tumor resistance, the reduction of side effects, and the optimization of dosage form for breast cancer treatment. At the same time, it is inevitable to find components like paclitaxel in plants such as *S. miltiorrhiza* that have significant therapeutic effects on breast cancer. Fortunately, Tan IIA was reported to be more effective than tamoxifen, which is the first-line drug for breast cancer (Lu et al., 2009).

Despite the progress in understanding the phytochemistry and the anti-breast cancer pharmacology of *S. miltiorrhiza*. There are still some issues that need deeper investigation. Firstly, most of the current studies have been performed at cellular levels, whilst few are based on models. The systematic evaluation has not been closely investigated. Second, the exploration of the anti-breast cancer mechanisms is still scarce. The molecular mechanism types are mainly limited to apoptosis and cell cycle, which is far from enough in-depth. For example, although CPT, DHT, Tan IIA, and Tan I can induce apoptosis in breast cancer, it only includes regulation of caspase and Bcl-2 family proteins. Whether they have other potential targeted proteins merits further study. In addition, there are few studies focused on breast cancer metastasis, especially in relation to an application of immunotherapy, which are a hot issue in recent years. Therefore, the clinical application of *S. miltiorrhiza* active ingredients against breast cancer still deserves further investigation.

## Abbreviations

ABC, ATP-binding cassette; ATA, acetyltanshinone IIA; CDK, cyclin-dependent kinase; CPT, cryptotanshinone; CSCs, cancer stem cells; DHT, dihydrotanshinone I; DOX, doxorubicin; FDA, Food and Drug Administration; GPER, G-protein-coupled estrogen receptor; HER2, human epidermal growth factor receptor 2; RA, rosmarinic acid; Sal A, salvianolic acid A; Sal B, salvianolic acid B; Sal C, salvianolic acid C; *S. miltiorrhiza*, *Salvia miltiorrhiza*; STS, sodium Tan IIA sulfonate; Tan I, tanshinone I; Tan IIA, tanshinone IIA; TNBC, triple-negative breast cancer; TNF, tumor necrosis factor.
